# Feasibility of a computerized clinical decision support system delivered via a socially assistive robot during grand rounds: A pilot study

**DOI:** 10.1177/20552076251339012

**Published:** 2025-04-30

**Authors:** Valentino Šafran, Urška Smrke, Bojan Ilijevec, Samo Horvat, Vojko Flis, Nejc Plohl, Izidor Mlakar

**Affiliations:** 1 Faculty of Electrical Engineering and Computer Science, University of Maribor, Maribor, Slovenia; 2 University Division of Surgery, University Medical Centre Maribor, Maribor, Slovenia; 3 Faculty of Arts, Department of Psychology, University of Maribor, Maribor, Slovenia

**Keywords:** Clinical decision support systems, clinical decision-making, hospital grand rounds, patient data integration, perceived quality of care, socially assistive robots, usability and familiarity, user experience questionnaire, workload reduction

## Abstract

**Aims and Objective:**

The aim of this study was to explore the feasibility, usability and acceptance of integrating Clinical Decision Support Systems with Socially Assistive Robots into hospital grand rounds.

**Background:**

Adopting Clinical Decision Support Systems in healthcare faces challenges such as complexity, poor integration with workflows, and concerns about data privacy and quality. Issues such as too many alerts, confusing errors, and difficulty using the technology in front of patients make adoption challenging and prevent it from fitting into daily workflows. Making Clinical Decision Support System simple, intuitive and user-friendly is essential to enable its use in daily practice to improve patient care and decision-making.

**Methods:**

This six-month pilot study had two participant groups, with total of 40 participants: a longitudinal intervention group (n = 8) and a single-session evaluation group (n = 32). Participants were medical doctors at the University Clinical Center Maribor. The intervention involved implementing a Clinical Decision Support System delivered via a Socially Assistive Robot during hospital grand rounds. We developed a system that employed the HL7 FHIR standard for integrating data from hospital monitors, electronic health records, and patient-reported outcomes into a single dashboard. A Pepper-based SAR provided patient specific recommendations through a voice and SAR tablet enabled interface. Key evaluation metrics were assessed using the System Usability Scale (SUS) and the Unified Theory of Acceptance, Use of Technology (UTAUT2) questionnaire, including Effort Expectancy, Performance Expectancy and open ended questions. The longitudinal group used the system for 6 months and completed the assessments twice, after one week and at the end of the study. The single-session group completed the assessment once, immediately after the experiment. Qualitative data were gathered through open-ended questions. Data analysis included descriptive statistics, paired t-tests, and thematic analysis.

**Results:**

System usability was rated highly across both groups, with the longitudinal group reporting consistently excellent scores (M = 82.08 at final evaluation) compared to the acceptable scores of the single-session group (M = 68.96). Extended exposure improved user engagement, reflected in significant increases in Effort Expectancy and Habit over time. Participants found the system enjoyable to use, and while no significant changes were seen in Performance Expectancy, feedback emphasized its efficiency in saving time and improving access to clinical data, supporting its feasibility and acceptability.

**Conclusions:**

This research supports the potential of robotic technologies to transform CDSS into more interactive, efficient, and user-friendly tools for healthcare professionals. The paper also suggests further research directions and technical improvements to maximize the impact of innovative technologies in healthcare.

## Introduction

### Background on CDSS and their importance in healthcare

Clinical Decision Support Systems (CDSS) have become an important tool in modern healthcare. Patient care now often relies on systems that provide recommendations tailored to specific patients using electronic health data. CDSS helps healthcare providers by combining data from sources like electronic health records (EHRs) with clinical guidelines and medical knowledge to support decision-making.

Clinicians face complex medical situations and must make quick decisions, and CDSS assist by offering fast and accurate insights.^1^ However, even with the growing use of CDSS, making decisions in healthcare is still very complicated, especially when there are many factors to consider, not enough time, or missing information.^2^

By giving real-time access to updated patient data and clear recommendations, CDSS support better decisions and help reduce medical errors, particularly in critical and complex cases.^3^ These systems improve the quality of care by ensuring clinicians have all the necessary information readily available.^4,5^

### Challenges in CDSS adoption and implementation

Despite their potential, adopting and implementing CDSS in healthcare systems faces many challenges. Some barriers include system complexity, poor integration into existing workflows, and concerns about data privacy and quality as stated below.

Many healthcare providers are hesitant to use CDSS because they find it complicated, worry about errors in decision-making, and feel overwhelmed by too many alerts or notifications. Privacy concerns and discomfort with using advanced technology in front of patients also limit its widespread use.

The complexity of the systems where many clinicians perceive CDSS as complex is leading to hesitance in usage. Studies indicate that user-friendliness is a significant factor affecting the acceptance of CDSS, with many clinicians expressing concerns about the system's complexity and usability,^3,6^ especially for the older expert cohorts.^7^

Another major challenge is poor integration into clinical workflows. Effective integration with existing EHRs and workflows is essential. Researches show that when CDSS is not well-integrated, it disrupts normal processes and increases the workload for clinicians, which discourages them from using the system.^6,8^

Data privacy and quality are major concerns for clinicians. Ensuring that patient information remains secure is essential for building trust among clinicians and patients, as well as for the successful implementation of CDSS.^3,8^ Using advanced technology in front of patients can create privacy concerns, which may discourage healthcare providers from fully using CDSS. Addressing these concerns is vital for fostering an environment conducive to the effective use of these systems.

Clinicians are often reluctant about relying on CDSS due to fears of potential errors in decision-making processes. This is a concern because getting too many alerts or notifications from the system could make clinicians feel worn out and lead to alert fatigue.^1,9^

Furthermore, some clinicians are also hesitant to adopt new technologies, especially in clinical settings where traditional methods are firmly established. This cultural resistance can hinder the widespread adoption of CDSS.^1,6^

Finally, a wide variety of studies on digital systems often involve single exposure assessments, primarily due to their simplicity, efficiency and streamlining of the research processes.^9,10^ While these studies provide immediate insights into how users interact with a product under specific conditions, they cannot account for changes in user experience or usability over time or the effects of repeated use.^11^ Namely, in studies of human-machine and human-robot interaction, particularly involving socially assistive robots (SARs), it has been observed that participants typically require multiple interactions to develop a sense of familiarity and habitual engagement with the robot.^12,13^ Moreover, single-exposure evaluations may overemphasize first-impression problems while missing critical aspects of long-term adoption.

This study addresses key challenges in adopting CDSS by improving usability, making integration with existing EHRs easier, and encouraging clinician engagement. User-friendly interfaces and streamlined workflows reduce complexity, making it easier to fit CDSS into daily clinical routines. Simplified notifications and repeated use help prevent alert fatigue, giving clinicians time to become comfortable with the system. The study also focuses on digitalizing and standardizing data to ensure consistency and make patient information easier to access. These improvements aim to make CDSS adoption smoother and more effective in real-world healthcare settings. To address these challenges and improve CDSS adoption, researchers have begun exploring innovative interfaces, including the use of SARs in healthcare settings.

### The role of socially assistive humanoid robots in healthcare

SARs are increasingly recognized for their potential to enhance both patient care and the operational efficiency of healthcare providers. Robots like Frida which is a Pepper robot can be and are already present in hospitals and can be used for real-time patient monitoring, improving communication among healthcare teams and acting as interactive interfaces for CDSS.^14^ This integration then streamlines decision-making processes and increases accuracy in clinical settings.^3^

SARs also collect vital data, such as blood pressure and heart rate, and offer treatment suggestions, making patient visits more efficient while improving the overall quality of care.^6^ Furthermore, the human-like interaction of robots like Frida fosters trust and engagement, which is essential for the successful adoption of robotic technology in healthcare environments. In addition to Frida, other robots like NAO and ASIMO have been used in healthcare.^15,16^ NAO has made pediatric physical therapy more interactive and engaging, while ASIMO assists patients with mobility issues during rehabilitation.^17^ Emotional support robots, such as Paro, have been particularly beneficial in elderly care, reducing feelings of loneliness and agitation among dementia patients through soothing interactions.^18^

Despite these advantages, challenges such as staff workload, privacy concerns, and the need for proper training in robotic systems must be addressed to fully realize their potential. As technology advances, robots are expected to take on more complex roles in healthcare, such as assisting in surgeries and diagnoses, highlighting their transformative potential in the future.

Using the Pepper robot, clinical monitors, and hospital systems, we gathered important data such as patient history, lab results, and vital signs. This helped clinicians and nurses make faster decisions and deliver better care. We also focused on making Pepper's interactions more natural to build trust and comfort for both doctors and patients. We observed and compared how the user perceptions and system effectiveness between group of clinicians with a single exposure to the system differed from those with long exposure.

Additionally, we addressed challenges like staff workload, privacy, and training, offering ways to integrate robots safely and effectively into daily care. To achieve this, we focused on making the system easy to use and by integrating it into clinicians’ workflows to reduce their workload. To ensure privacy, we implemented strong data security to keep patient information safe. We also provided short training to help clinicians feel confident and comfortable using the technology. By tackling these issues, our research makes it easier to bring robots into healthcare and unlock their potential to improve care. This research demonstrates how SARs could play a larger and more valuable role in the future of healthcare.

### Study objectives and hypotheses

This study aimed to evaluate the feasibility and acceptability of implementing a CDSS delivered through a SAR during medical Grand Rounds by examining both technical usability and user acceptance factors in a clinical setting as represented in Table 1. To do this, we set the following research questions: (1) “Is it feasible and acceptable to implement a robot-delivered CDSS during medical Grand Rounds?”, and (2) “How do user perceptions and system effectiveness differ between single exposure and longitudinal use?”. We hypothesize that clinicians will accept (H1) and find the CDSS useful (H2). Moreover, we hypothesize that integration will be perceived as more effective after longitudinal exposure compared to single exposure (H3).

**Table 1.** Study objectives and hypothesis.

Objective	Description	Related hypothesis
Usability Assessment	Evaluate the usability of the CDSS-SAR system in clinical settings	H1: The CDSS-SAR system will demonstrate high usability scores (SUS > 68) among healthcare professionals
User Acceptance	Assess healthcare professionals’ acceptance of the CDSS-SAR system	H2: Healthcare professionals will show positive acceptance of the CDSS-SAR system as measured by UTAUT2 constructs
Longitudinal Impact	Investigate changes in usability and acceptance over time	H3: Usability and acceptance scores will improve significantly over the six-month study period
Workflow Integration	Evaluate the system's integration into clinical workflows	H3: The CDSS-SAR system will be perceived as well-integrated into existing clinical workflows
Perceived Benefits	Assess perceived benefits in clinical decision-making and patient care	H3: Healthcare professionals will report improved decision-making and patient care quality when using the CDSS-SAR system

## Methods

### Study design

The study was designed to test the system's feasibility by comparing the single-session evaluation group with the longitudinal intervention group. For longitudinal intervention group, data were collected after the first week of grand rounds and again after the final grand round. Grand Round was formal meeting where medical cases were presented to an audience of doctors, residents, and medical students for educational purposes. The Final Grand Round was the last scheduled grand round session of the study period, occurring in May 2024. For single-session evaluation group, data were collected right after their single grand round session. The study took place over six months from June 2023 to May 2024 at the University Clinical Center Maribor, in the Thoracic, Abdominal, and General Surgery Departments, using a dedicated trial room.

### Participants

#### Longitudinal intervention group

Participants aged 18 years or above, who were employed as medical doctors at the university clinical center where the study took place, and were willing to participate in additional grand round sessions and subsequent evaluation, were invited to participate in the study. Overall, eight doctors were recruited to participate throughout the intervention phase of the study, with a male-to-female ratio of 3:1. The average age of participants was 32.5 years (*SD* = 2.78), ranging from 29 to 37 years. The average work experience was 6.5 years (*SD* = 3.21), with participants working in various departments, from thoracic (n = 5, 63,5%), abdominal and general (n = 1, 12.5%), vascular (n = 1, 12.5%), and neurology surgery (n = 1, 12.5%) units. The cohort initially comprised eight participants; however, two participants (25%) dropped out before completing the final questionnaire. Notably, the dropouts were both male. The comparison of dropout rates by department showed that clinicians from the Vascular and Neurology departments stopped participating, suggesting potential department-specific challenges in sustained participation.

#### Single-session evaluation group

In the single-session experiment, we, again, aimed to recruit participants aged 18 years of above, who were employed as medical doctors at the university clinical center, and willing to participate in one grand round session and subsequent evaluation. 32 doctors with a mean age of approximately 47.8 years (*SD* = 10.4 years) participated, with a 59% of subject identifying themselves as male and 41% participants as females. The cohort involved doctors from Oncology-Related Departments (n = 8, 28.1%), Surgical Departments (n = 6, 18.8%), Internal Medicine Departments (n = 5, 15.6%), Emergency and Intensive Care (n = 3, 9.7%), Pediatric Departments (n = 2, 6.5%) and Other Clinical Departments (n = 8, 28.1%).

#### Inclusion and exclusion criteria

For both the longitudinal intervention group of medical doctors and the single-session evaluation group, the following inclusion criteria were applied: age 18 years or above, employed as a medical doctor at the University Clinical Center Maribor, willing to participate in the study and provide informed consent. Participants were excluded from the study if they met any of the following criteria: unexpected important procedures that could interfere with the ability to use the CDSS or complete questionnaires, unwilling or unable to comply with the study protocol.

### Intervention

**Figure 1.**
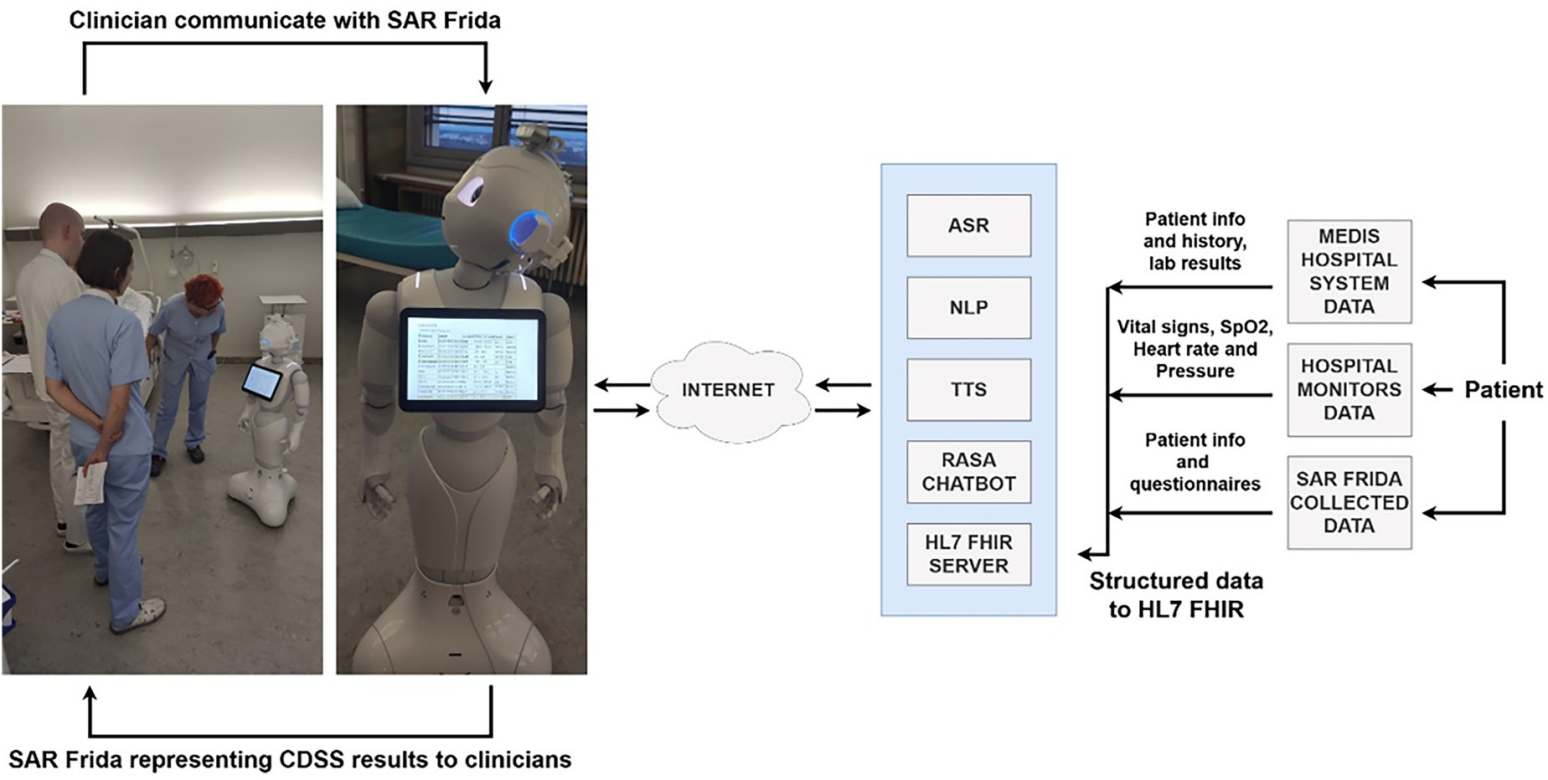
Implementation and delivery of intervention.

The CDSS developed for this study leverages the Health Level Seven Fast Healthcare Interoperability Resources (HL7 FHIR) standard to integrate various patient data sources. This system is coupled with a Socially Assistive Robot (SAR), Pepper model named Frida as represented on Figure 1, to create an intuitive interface that assists clinicians during grand rounds. The CDSS provides real-time clinical decision support, offering patient-specific recommendations based on both historical and real-time health metrics. The humanoid robot Frida serves as a facilitator of data collection, visualization, and interaction, allowing the healthcare team to access and act upon critical information efficiently. Frida communicated over representational state transfer application programming interface (REST API) with microservices Automatic Speech Recognition (ASR), Text to Speech (TTS), Natural Language Processing (NLP), Rasa Chatbot. ASR provides the speech recognition in the flow between the communication of the clinicians and the SAR, while the TTS is used to give back a response from the SAR to clinicians. Rasa Chatbot was used in the process of retrieving the information from the patients which was inserted in the Health Level Seven Fast Healthcare Interoperability Resource (HL7 FHIR) and used in the CDSS to provide additional information about the patient to clinicians.

The intervention focused on the feedback and responses collected from clinicians who utilized the Frida robot as part of the study's CDSS during hospital grand rounds. The participating clinicians interacted with the system, which was designed to enhance both efficiency and accuracy in clinical decision-making processes. The Frida robot, equipped with sensors and cameras, served as an interface between clinicians and patients, presenting patient data, offering clinical recommendations, and collecting additional inputs in real time.

Throughout the intervention phase, clinician feedback was systematically gathered via self-report questionnaires, including the System Usability Scale (SUS) and the Unified Theory of Acceptance and Use of Technology (UTAUT2) instruments. These questionnaires assessed the usability, efficiency, and perceived utility of the robotic platform. The initial assessment was after the first week of grand rounds. The final assessment was after the last grand round in May 2024. Longitudinal participants attended weekly grand rounds, with a minimum of one and up to three grand rounds for a patient on the same day. On the day of the grand round, one to two patients were included. Each grand round lasting approximately 5–10 min, where we used the CDSS with SAR system during each session. Participants that participated in a single grand round session had one assessment at the end of the study period in May 2024. The assessment was immediately following their single session. Participants attended one grand round session lasting 30–40 min and we used CDSS with SAR system.

The HL7 FHIR framework was chosen for its flexibility and robust interoperability. HL7 FHIR supports the integration of diverse healthcare data formats such as EHRs, real-time health monitoring, and patient-reported outcomes (PROs). Through its modular resource structure and REST API, FHIR enables seamless data exchange between various systems, ensuring that the CDSS has access to complete and accurate patient information. By standardizing this data exchange, FHIR reduces compatibility issues typically encountered when trying to integrate different healthcare systems, leading to improved care continuity and clinical decision-making. In this system, the key resources used included:

- Patient Resource: Containing demographic data, medical history, and clinical encounters.
- Observation Resource: Tracking vital signs such as heart rate, blood pressure, and temperature, crucial for real-time monitoring.
- DiagnosticReport Resource: For accessing lab results and other diagnostics essential for decision-making during rounds.
- Composition Resource: Compiles detailed clinical notes, non-standard questionnaire results ensuring a comprehensive view of patient encounters and supporting continuity of care.
- QuestionnaireResponse Resource: Captures structured responses to questionnaires completed by patients or clinicians, facilitating the collection of standardized patient-reported data.

The FHIR-based CDSS utilized REST APIs to integrate with hospital systems and external devices (e.g., clinical monitors). This enabled real-time updates of patient conditions, providing clinicians with a holistic view of each patient's status during rounds. Additionally, the FHIR server allowed for interoperability, making it possible to exchange patient data between different healthcare systems.

### Implementation during the grand round routine

**Figure 2.**
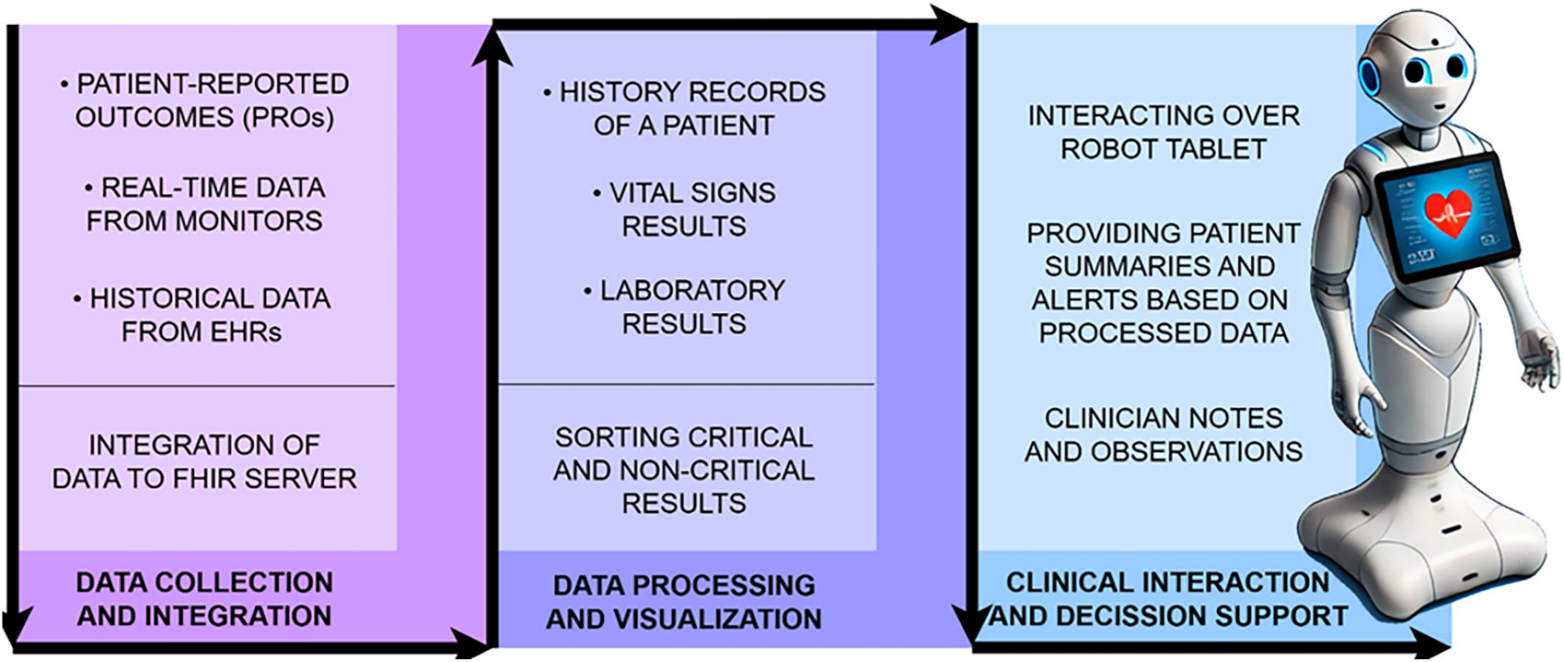
Data flow of the intervention.

The system was implemented during hospital grand rounds to enhance clinical efficiency and decision accuracy. Frida, the SAR, acted as a mobile platform for interacting with the CDSS. The Frida robot was equipped with sensors and cameras to assist in capturing patient data and facilitating real-time interactions between clinicians and patients. During grand rounds, Frida was responsible for presenting patient data, offering clinical recommendations from the CDSS, and collecting additional data through direct patient interaction. The workflow of the system involved several stages of data integration, visualization, and interaction. Figure 2 illustrates the data flow for intervention.

The visit was conducted as an additional session, following an initial visit performed by the clinicians in the study. Before the visit, we imported data from monitors and medical information system (MEDIS). Monitor data included vital signs such as ECG heart rate, SpO2 saturation, SpO2 heart rate, and temperature. Data from MEDIS included general patient information, history, consultation details, clinic records, surgery descriptions, diagnoses, and laboratory results. Laboratory tests covered hematology, biochemistry, coagulation, proteins, tumor markers, and thyroid swabs. Not all MEDIS data categories apply to every patient; the information varies based on their past and current complications and procedures. Both data from monitors and MEDIS was converted and stored in FHIR format. During the visit, this data was displayed on a tablet. The robot first highlighted alarm situations but also allowed for intuitive data searches using speech. Doctors review the displayed data as part of the visit, enhancing their ability to assess the patient's condition.

The first clinical validation of the CDSS took place after the first week of use, and the final validation was conducted at the end of the study. Clinicians with long-term exposure who validated the CDSS after the first week also participated in the final validation. At the end of the study, a new group of clinicians, who were experiencing the system for the first time, was introduced to the CDSS and Frida. They were given the opportunity to try the system and were then asked the same as the long-term exposure group to complete the SUS and UTAUT2 questionnaires, along with a short qualitative assessment, to provide feedback.

### Validation measures

#### System usability scale (SUS)

The System Usability Scale (SUS) is a widely recognized tool used to evaluate the usability of various systems, including software, hardware, and applications.^19^ The SUS questionnaire includes ten statements that respondents rate on a scale from 1 (Strongly Disagree) to 5 (Strongly Agree). The statements cover various aspects of usability, such as Ease of Use, Complexity, Learnability, Confidence, Integration, Support and Satisfaction. Although SUS was not specifically designed to evaluate digital health technologies, the widely accepted benchmark of a mean SUS score of 68 (SD 12.5) is suitable for evaluating their usability.^20^

#### Unified theory of acceptance and use of technology (UTAUT/UTAUT2) questionnaire

The Unified Theory of Acceptance and Use of Technology (UTAUT) and its extension, UTAUT2, are frameworks designed to understand the factors influencing user acceptance and usage of technology.^21,22^ Both models provide a comprehensive framework for understanding user acceptance of technology by examining multiple dimensions of user experience that can help predict behavioral intentions toward adopting new technology, e.g., Performance Expectancy, Effort Expectancy, Social Influence and Facilitating Conditions. The UTAUT2 builds on the original UTAUT model by extending it with three additional constructs, Hedonic Motivation, Price Value and Habit. While again not specifically designed for medical applications, UTAUT2 is well-suited for predicting the acceptance of mHealth applications due to its focus on individual user experiences and motivations.^23^

#### Open-ended questions

To further understand healthcare professionals’ responses we carried out qualitative assessment as part of the comprehensive evaluation of the proposed healthcare technology solution. At the end of the single session group and in the final measurement of the longitudinal group we asked them three questions:

AQ1: Please give your opinion on what you think about the proposed solution that you had the opportunity to see during the visit (General Perceptions)
AQ2: Please indicate the weaknesses that you have observed in the proposed solution that you had the opportunity to see during the visit. (Implementation Challenges)
AQ3: Please indicate the improvements you would suggest and other notes and things that would be useful and helpful to you during your visits (Development Opportunities).

### Patient data collection

**Figure 3.**
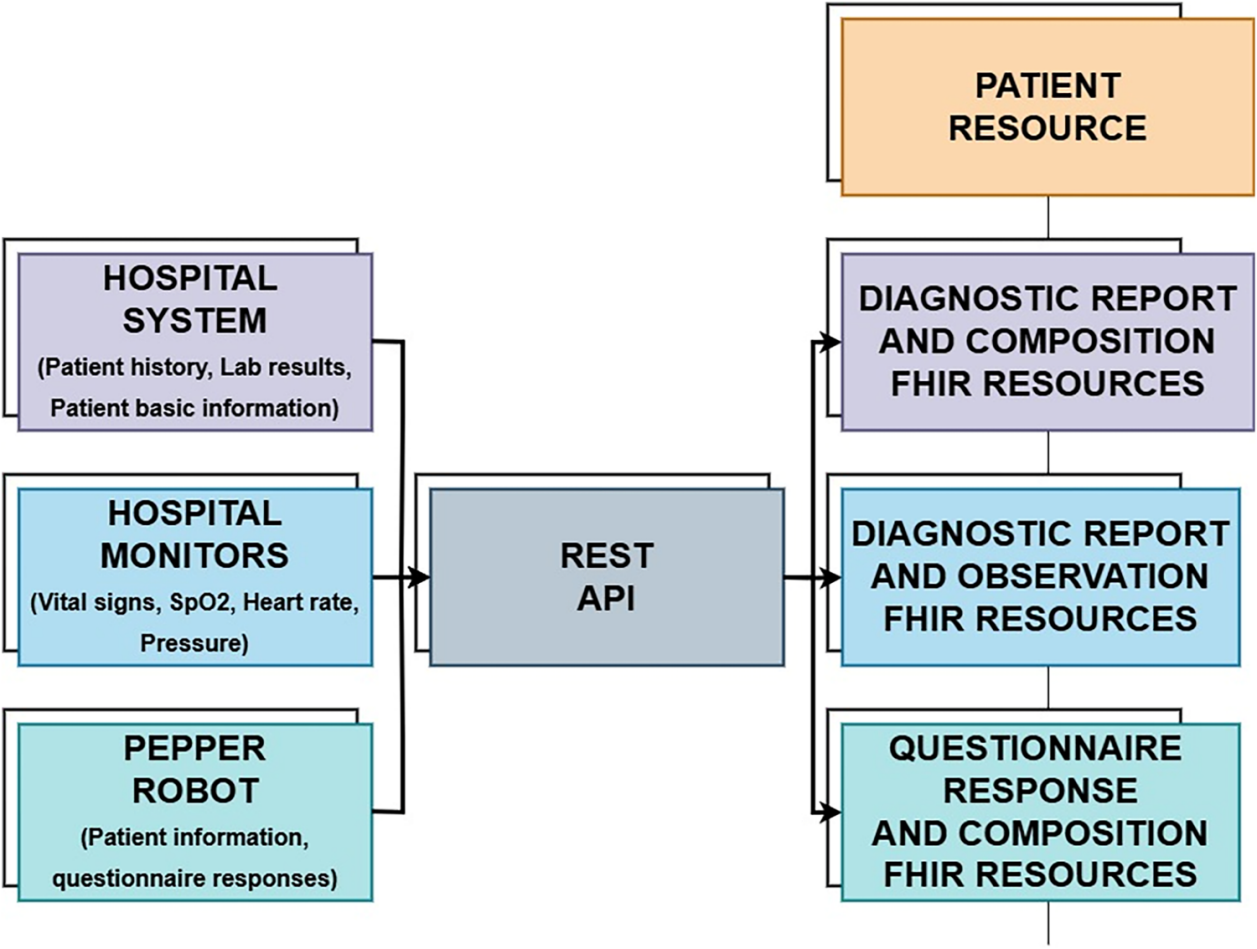
Sources of patient data and mapping to the FHIR resources.

The system's workflow began with a comprehensive data collection process as represented in Figure 3. Real-time data from four Mindray monitors, connected to a central station via Wi-Fi, continuously gathered vital metrics such as temperature, oxygen saturation (SpO2), and heart rate. This information was synchronized with the FHIR server to ensure that the clinical team received up-to-date data. The patient data from hospital IT system were then imported for all included patients in the study where the newest added data together with all the patient history and laboratory results was converted to the FHIR standard into the appropriate FHIR resources. Lastly, the patient-reported outcomes PROs collected by the Frida were summarized and represented to the clinicians as the extra information. They were being able to see it as a part of CDSS. Data for this study was collected from 229 patients.

#### Patient data from healthcare monitors

In the study, each patient had to agree to be connected to the clinical monitor sensors to gather the temperature, oxygen saturation (SpO2), and heart rate. Patients that participated in the monitor data collection had to wear the monitor sensors for at least a few hours periods a few times a day. Monitors had the Wi-Fi connection to the monitor main central station where the collected data was stored in a HL7 FHIR format. Over secured REST API and firewall protected tunnel, data from monitors was accessed remotely, then extracted from the monitor FHIR database and processed to be represented on a CDSS dashboard to clinicians. As a result, clinicians were able to see the tabular numerical values and graphical trends of those values to see if there were any abnormalities in collected patient vital signs.

#### Patient data collected by SAR

Additionally, questionnaires responses were collected through Frida speech-enabled interface, which allowed patients to communicate their pain levels and psychological distress. These responses were logged immediately in the FHIR database as QuestionnaireResponse and Composition resources. Historical data from electronic health records (EHRs), including past medical history, diagnoses, and lab results, was also imported into the system. This data was transformed into FHIR-compatible format as Composition resource and made accessible on the FHIR dashboard.

#### Patient data from hospital it system

First, demographic information, such as age, gender, and medical identification, was imported as FHIR Patient Resources, allowing personalized recommendations that clinicians could easily access. Medical history and past diagnoses, including chronic conditions and surgeries, were converted into FHIR Condition Resources, enabling clinicians to review previous medical events and assess their relevance to current care. Previous treatments, surgeries, or hospitalizations were organized as Composition Resources, giving insight into each patient's health progression.

Laboratory results and diagnostic reports, including blood glucose levels, and other crucial markers, were imported as DiagnosticReport and Observation resources. Abnormal values were flagged to help clinicians identify health trends, such as a history of high cholesterol indicating cardiovascular risks. Additionally, historical medication records were imported, allowing the CDSS to check for medication interactions and promote safe prescribing practices. Known allergies were also included to alert clinicians of potential risks in treatment plans.

### Study measures data collection

In addition to the clinical data collected for the CDSS, we gathered data specifically for the validation measures described in Section 2.5. Regarding SUS, participants completed the 10-item questionnaire after their interaction with the system. For the longitudinal group, SUS data were collected at two time points: after the first week of use and at the end of the study period. The single-session group completed the SUS once, immediately after their interaction with the system. For UTAUT2 participants filled out the questionnaire, which assessed the seven constructs: Performance Expectancy, Effort Expectancy, Social Influence, Facilitating Conditions, Hedonic Motivation, Habit, and Behavioral Intention. The longitudinal group completed the UTAUT2 at the same two time points as the SUS. The single-session group completed the UTAUT2 once, following their system interaction. Regarding open-ended questions, qualitative data was collected through three open-ended questions (AQ1, AQ2, AQ3) as described in Section 2.5.3. These questions were administered to both groups at the end of their respective study periods. All questionnaires were administered electronically through a secure, web-based survey platform. Responses were anonymized and linked to participant IDs for analysis. The data collection process was designed to minimize disruption to clinical workflows while ensuring comprehensive evaluation of the system's usability and acceptance.

### Statistical analysis

Quantitative data from the SUS and UTAT2 instruments were analyzed using Python libraries, specifically pandas, numpy, scipy, pingouin, matplotlib and seaborn. Statistical analyses were performed with pandas for data manipulation and scipy.stats for statistical testing. Descriptive statistics, including means and standard deviations, were calculated for all constructs and components. Missing values (<5% of data) were handled using mean imputation to maintain the sample size while minimizing impact on the analyses. For the UTAUT2 analysis, we calculated construct scores by averaging the corresponding items for each construct (Performance Expectancy, Effort Expectancy, Social Influence, Facilitating Conditions, Hedonic Motivation, Habit, and Behavioral Intention). SUS scores were analyzed as a single score with scores converted to a 0–100 scale following standard SUS scoring procedures.

For group comparisons, we analyzed three evaluation approaches: longitudinal start (*n* = 6), longitudinal end (*n* = 6), and single-session exposure (*n* = 32). Paired t-tests were used to compare scores between start and end measurements (*n* = 6) for both UTAUT2 constructs and SUS components. Statistical significance was set at *p* < .05. Effect sizes were calculated to quantify the observed differences and relationships, regardless of statistical significance. The analysis in the relationships between demographic variables (age, gender, work experience) and both UTAUT2 constructs and SUS components were analyzed using Pearson correlations for continuous variables.

Qualitative data from open-ended questions were analyzed using data-driven thematic analysis to evaluate healthcare professionals’ experiences and perceptions of the SAR solution through qualitative feedback.^24^ The analysis focused on three key areas: general perceptions, identified weaknesses, and suggested improvements. Responses to three open-ended questions were reviewed and initial open coding was performed to categorize meaningful segments of text, followed by the classification of results into categories, sub-categories, and specific themes. Representative quotes were selected to illustrate key themes, and frequency counts were calculated to determine the relative prominence of identified patterns. The process was supported by Python's natural language processing library NLTK (Natural Language Toolkit), to assist in text preprocessing and frequency analysis of key terms.

## Results

### Longitudinal intervention group

The analysis revealed complementary findings represented in Table 2 across both UTAUT2 and SUS measurements. The UTAUT2 analysis showed notable improvements in Hedonic Motivation and Habit, indicating increased enjoyment and system integration into clinical routines. The positive user experience was strongly corroborated by the consistently excellent SUS scores, which remained high throughout the study period. While Performance Expectancy and Effort Expectancy showed a minimal decrease, both maintained high scores throughout. Social Influence showed a moderate decrease, suggesting reduced dependence on others’ opinions over time, while Facilitating Conditions also showed a slight decline. Also, Behavioral Intention maintained strong scores despite a small decrease, indicating sustained willingness to use the system. Table 2 presents the results and correlations between first iteration representing scores after first week of use and final iteration representing scores after last grand round.

**Table 2.** Descriptive statistics, reliability, and correlations for UTAUT2 and SUS constructs in longitudinal intervention group, two measurements.

Scale/Construct	First iteration	Final iteration	Change
	*M(SD)*	*M(SD)*	
Performance Expectancy	4.11 (0.46)	4.00 (0.67)	*t*(5) = .38, *p* = .721, *d* = −.19
Effort Expectancy	4.33 (0.52)	4.28 (0.57)	*t*(5) = .21, *p* = .842, *d* = −.10
Social Influence	3.39 (0.49)	2.89 (1.05)	*t*(5) = 1.17, *p* = .296, *d* = −.61
Facilitating Conditions	3.94 (0.39)	3.67 (0.63)	*t*(5) = 1.05, *p* = .341, *d* = −.53
Hedonic Motivation	3.72 (0.39)	4.17 (0.46)	*t*(5) = −2.17, *p* = .082, *d* = 1.04
Habit	3.06 (0.14)	3.56 (0.81)	*t*(5) = −1.42 *p* = .215, *d* = .86
Behavioral Intention	4.33 (0.52)	4.17 (0.46)	*t*(5) = 1.00, *p* = .363, *d* = −.34
System Usability	84.58 (10.42)	82.08 (13.91)	*t(5*) = 1.00, *p* = .363*, d* *=* *−.20*

*Note.* * *p* < .050, ^**^ *p* < .010., First Iteration – represents scores collected after first week of use, Final Iteration – represents scores collected after the last grand round performed.

We have also analyzed the correlations between the UTAT2 constructs and the variables of age, gender, and work experience. While in the first iteration, none of the correlations reach statistical significance (p < 0.05), the Habit (r = 0.88, p = 0.022) with behavioral intention (r = 0.77, p = 0.073) work experience is nearing the significance. This suggests that while relationships may exist, they are not strong enough to be conclusive with the current sample.

### Single-session evaluation group

The single-session based evaluation demonstrated findings further supporting the evaluation by the longitudinal group, with strong acceptance and usability across all constructs as reported in Table 3. The UTAUT2 scores across all constructs, suggesting an overall positive response to the robot after observing a single interaction. Notably, Hedonic Motivation scored the highest, indicating that participants found interacting with the robot enjoyable, a critical factor in promoting engagement and encouraging future use. Meanwhile, Performance Expectancy and Effort Expectancy were also moderately rated, suggesting that participants perceived the robot as potentially helpful and reasonably easy to use, though the scores reflect room for improvement in these areas to further boost acceptance. The relatively low score in Habit suggests that participants have not yet developed a habitual relationship with the socially assistive robot, which is expected given that this was a single-exposure scenario. The SUS score, with an average of 68.96, is above the standard threshold of 68, which typically signifies acceptable usability. Similarly, as for Habit, the lower score is somewhat expected since a single exposure may not give users enough time to fully explore or adapt to the system. Table 3 summarizes the results and correlations between age, gender and work experiences with UTAUT2 and SUS constructs.

**Table 3.** Descriptive statistics, and correlations for UTAUT2 and SUS constructs in single-exposure group, single measurement.

	Total sample	Gender		Correlations		
Scale/Construct	*M* (*SD*) (n = 31)	Male (*n* = 18)	Female (*n* = 13)	Gender (p)	Age (*p*)	Experience (*p*)
Performance Expectancy	3.45 (.99)	3.59 (0.95)	3.31 (1.12)	.277 (.132)	−0.138 (0.460)	−0.097 (0.604)
Effort Expectancy	3.58 (.77)	3.70 (0.77)	3.62 (0.89)	.152 (.415)	−0.044 (0.815)	−0.008 (0.965)
Social Influence	3.10 (.69)	3.15 (0.75)	3.03 (0.82)	.108 (.564)	0.222 (0.229)	0.222 (0.231)
Facilitating Conditions	3.21 (.61)	3.30 (0.90)	3.33 (0.91)	−.293 (0109)	−0.224 (0.226)	−0.296 (0.106)
Hedonic Motivation	3.95 (.87)	4.04 (0.87)	3.87 (0.94)	−.101 (.589)	0.221 (0.232)	0.162 (0.383)
Habit	2.72 (.54)	2.76 (0.73)	2.64 (0.69)	.206 (.267)	0.223 (0.227)	0.212 (0.253)
Behavioral Intention	3.82 (.67)	3.83 (.84)	3.69 (0.88)	.248 (.179)	0.200 (0.281)	0.189 (0.310)
	(n = 32)	Male (*n* = 19)	Female (*n* = 13)			
System Usability	68.96 (9.72)	68.81 (9.09)	69.19 (10.96)	−.020 (.916)	.038 (.838)	0.058 (.753)

*Note.* M = Mean; SD = Standard Deviation;. All constructs measured on a 5-point Likert scale (1 = strongly disagree to 5 = strongly agree). p indicates if the scores are statistically different between genders, age and work experience (* = *p* < 0.05,** *p* < 0.01); Age range = 25–69 years; Experience = 0–39 years; For UTAUT2 one participant didn’t answer all questions and was discarded. This is why n = 31 in first part of table and n = 32 in second part.

The results indicate that perceptions of the system's usability and various expectancy constructs, including performance and effort expectancy, social influence, facilitating conditions, hedonic motivation, and habit, are consistent across genders, as well as age and experience levels. The lack of significant gender differences and weak correlations with age and experience suggest that the system is perceived similarly by both male and female users and is not strongly influenced by demographic factors. This uniformity in responses may reflect a design that is broadly accessible and user-friendly, accommodating a wide range of users without significant variance in their intention to use or satisfaction with the system.

### Comparison between the groups

To better understand the differences between the longitudinal and single-session groups, we conducted a comparative analysis. Table 4 and Table 5 present the results of this comparison, highlighting the differences in UTAUT2 and SUS constructs between the groups at different time points.

**Table 4.** Descriptive correlations for UTAUT2 and SUS constructs between first measurement of the longitudinal intervention group and single-exposure group.

Scale/Construct	Single-session evaluation Group	Longitudinal intervention group, First measurement	Final vs Single	
	M (SD)	M (SD)	t	p
Performance Expectancy	3.45 (.99)	4.11 (.46)	1.58	.123
Effort Expectancy	3.58 (.77)	4.33 (.52)	2.31	.027*
Social Influence	3.10 (.69)	2.81 (1.05)	.97	.337
Facilitating Conditions	3.21 (.61)	3.94 (0.63)	2.81	.008**
Hedonic Motivation	3.95 (.87)	3.72 (0.46)	−.61	.545
Habit	2.72 (.54)	3.06 (0.81)	1.50	.143
Behavioral Intention	3.82 (.67)	4.33 (0.46)	1.78	.084
System Usability	68.96 (9.72)	84.58 (10.42)	3,75	.001**

*Note.* M = Mean; SD = Standard Deviation, * = *p* < 0.05,** *p* < 0.01, higher scores indicate better outcomes across all measures.

**Table 5.** Descriptive correlations for UTAUT2 and SUS constructs between last measurement of the longitudinal intervention group and single-exposure group.

Scale/Construct	Single-session evaluation group	Longitudinal intervention group, Final measurement	Final vs Single	
	M (SD)	M (SD)	t	p
Performance Expectancy	3.45 (.99)	4.00 (.67)	1.29	.205
Effort Expectancy	3.58 (.77)	4.28 (.57)	2.12	.041*
Social Influence	3.10 (.69)	2.89 (1.05)	−.63	.531
Facilitating Conditions	3.21 (.61)	3.67 (.63)	1.66	.105
Hedonic Motivation	3.95 (.87)	4.17 (.46)	.60	.554
Habit	2.72 (.54)	3.56 (.81)	8.943	.003 **
Behavioral Intention	3.82 (.67)	4.17 (.46)	1.21	.233
System Usability	68.96 (9.72)	82.08 (13.91)	2.38	.008**

*Note*. M = Mean; SD = Standard Deviation, * = *p* < 0.05,** *p* < 0.01, higher scores indicate better outcomes across all measures.

We additionally compared the UTAUT2 and SUS variables between the first (Table 4) and final (Table 5) measurement in the longitudinal intervention group and the single-session group. While, the results of the first sessions measurement are quite similar to first time exposure in single session group and the final measurement represents a mature, stabilized user experience that better reflects actual system integration and effectiveness.^25,26^ The comparison of UTAUT2 and SUS constructs between single-exposure and longitudinal intervention groups reveals that repeated exposure positively impacts perceptions of system usability and ease of use. Specifically, the longitudinal intervention group reported significantly higher Effort Expectancy, Facilitating Conditions, and System Usability in the initial measurement, with Effort Expectancy and System Usability maintaining significance over time. By the final measurement, Habit also showed a significant increase, suggesting that regular use fosters familiarity and routine. Finally, the constructs such as Performance Expectancy and Behavioral Intention showed higher scores for the intervention group, these were not statistically significant. Overall, the results indicate that ongoing exposure enhances usability perceptions and encourages habitual system use, potentially supporting long-term adoption.

### Qualitative responses

**Figure 4.**
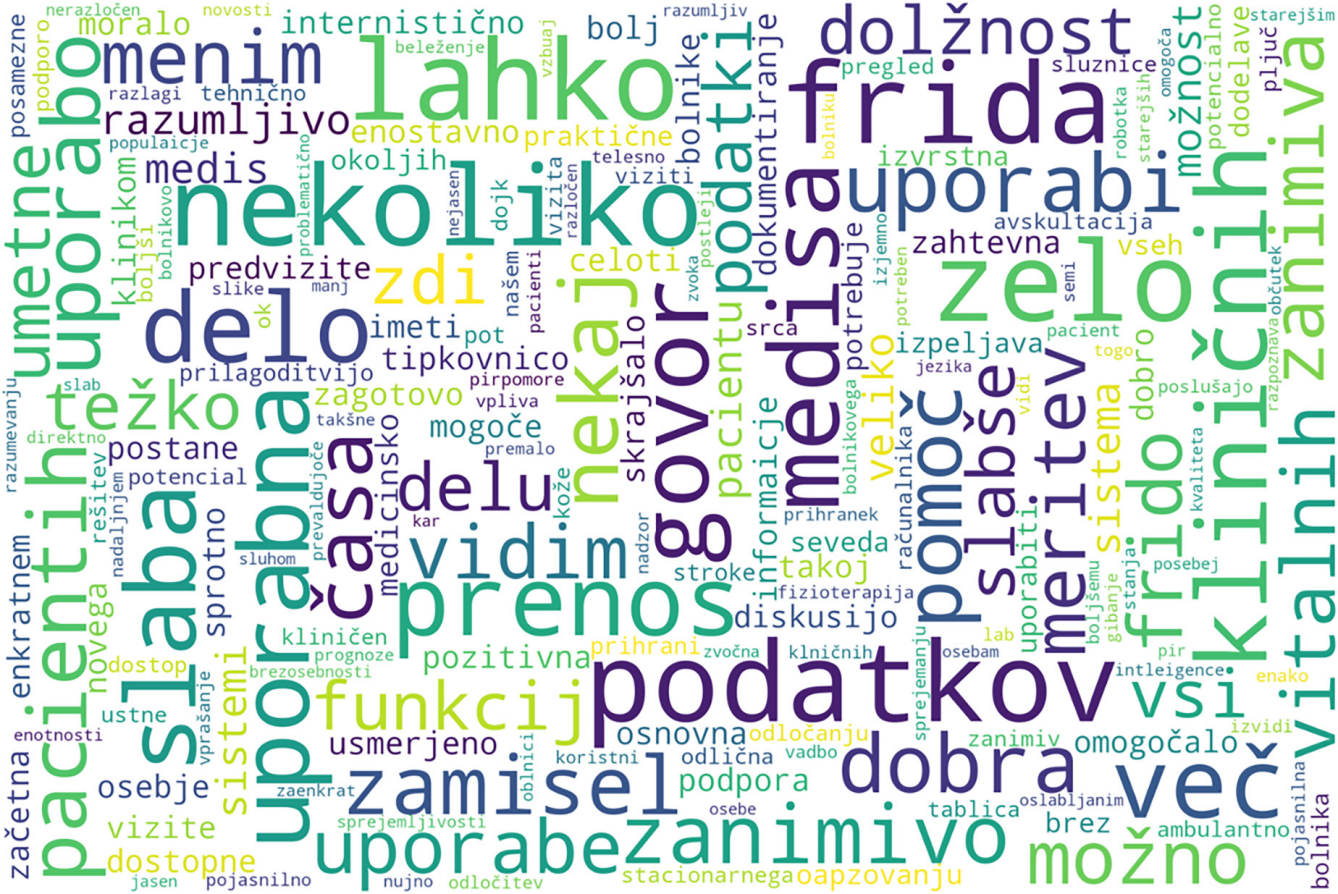
World cloud of qualitative responses (in slovenian).

Out of 32 participants in the Single-Session Evaluation Group, 24 participants responded, 65% to all 3 questions, an additional 30% to 2 questions and 5% to only 1 question. The word cloud is outlined in Figure 4.

The positive responses include recognition of SARs contribution to Efficiency & Time Management (e.g., “Shortens pre Q-visits and visits”, “Provides more time for medical discussions”, “Saves time by avoiding trips to stationary computers”), Access to Clinical Data (e.g., “Good access to clinical information”, “Useful for viewing laboratory results”) and Versatility (e.g., “Potential for both inpatient and outpatient use”, “Useful for physical therapy and monitoring”). The data was also analyzed using inductive thematic analysis. The results are summarized in Table 6.

**Table 6.** Analysis of qualitative responses.

Question	Category	Sub-Category	Theme	Representative quotes	N
AQ1	System Performance	Usability	Ease of Use	“Enostavno za uporabo (EN: “Easy to use”)	8
		Functionality	Information Accessibility	“Informacije dostopne takoj in v celoti” (EN: “Information available immediately and in full”)	7
	Clinical Impact	Support Features	Clinical Assistance	“Zagotovo v pomoč klinikom” (EN: “Definitely helpful to clinicians”)	6
		Specialization	Specialty-Specific Needs	“Mogoče bolj internistično usmerjeno” (EN: “Perhaps more internally medicine-oriented”)	4
	Patient Care	Time Efficiency	Visit Optimization	“Skrajšalo predvizite in vizite, omogočalo več časa za medicinsko diskusijo” (EN: Shortened pre-visits and visits, allowing more time for medical discussion	5
		Patient Education	Information Sharing	“Pojasnilna dolžnost: pacient vidi slike, vsi pacienti poslušajo enako pojasnilno dolžnost” (EN: “The patient sees images, all patients listen to the same informed consent”)	4
		Physical Activity	Exercise Support	“Robotka Frida vpliva tudi na bolnikovo telesno vadbo” (EN: “Robot Frida also influences patient's physical exercise”)	3
	Clinical Utility	Decision Support	Clinician Assistance	“Zagotovo v pomoč klinikom” (EN: “Definitely helpful to clinicians”)	7
		Workflow Optimization	Medical Discussion Time	“Omogočalo več časa za medicinsko diskusijo” (EN: “Allowed more time for medical discussion”)	6
		Staff Usability	Practical Applications	“Lahko postane uporabna tudi za osebje” (EN: “Can also become useful for staff”)	5
AQ2	Technical Issues	Interface	System Movement	“Nekoliko togo gibanje” (EN: “Somewhat rigid movement”)	7
		Integration	Patient Data Availability	“Potreben je prenos več podatkov iz Medisa” (EN: “Data transfer of more data from Medis is required”)	6
	User Concerns	Patient Acceptance	Care Process Impact	“Vprašanje sprejemljivosti takšne novosti pri starejših pacientih” (EN: “Question on acceptability of such a novelty among elderly patients”)	8
		Documentation	Bedside Documentation	“Beleženje direktno ob postelji nujno” (EN: “Recording directly at the bedside is essential”)	5
	Patient Care	Data Access	Patient Data Availability	“Niso dostopni vsi podatki o pacientu iz programa Medis” (EN: “Not all patient data from the Medis program is accessible”)	6
	Clinical Utility	AI Limitations	Current AI Capabilities	“Ne omogoča razširjenje uporabe umetne inteligence (zaenkrat)” (EN: “Does not allow the use of artificial intelligence (for now)”)	3
		Complex Cases	Case Adaptability	“Težja prilagodljivost zapletenih primerov” (EN: “Difficult transfer to complex cases”)	4
		Infection Control	Sterility Concerns	“Prenos okužb - sterilnost robota ob uporabi?” (EN: “Transmission of infections - robot sterility during use?”)	2
AQ3	Enhancement Requests	Technical	Interface Optimization	“Optimizacija povezave” (EN: “Connection optimization”)	6
		Language	Clarity and Translation	“Razločnejši jezik” (EN: “Clearer language”)	7
	Innovation	AI Integration	AI Implementation	“Uvedba več AI” (EN: “Integration of more AI”)	4
	Patient Care	Communication	Language Support	“Na voljo v jezikih naše populacije pacientov” (EN: “Available in all of the languages of our patient population”)	4
		Data Integration	Patient Records	“Povezava s CRPP (Centralni register podatkov o pacientu)” (EN: “Connection with Central Patient Data Register”)	3
	Clinical Utility	Research Applications	Clinical Trials Support	“Olajšalo tudi delo v samih kliničnih raziskavah, kjer jo vidim kot nekekšnega študijskega koordinatorja” (EN: “Would also facilitate work in clinical trials, where I see it as a kind of study coordinator”)	4
		Non-Medical Functions	Additional Informational Support	“Možnost podajanja tudi ne-medicinskih informacij (vreme itd.)” (EN: Possibility of providing non-medical information (weather, etc.)	2

Related to General Perceptions (i.e., “AQ1”) the analysis reveals a predominantly optimistic narrative, with moderately positive language. “Interesting” and “good” emerge as frequent descriptors, suggesting an overall positive reception. The term “useful” appears consistently, often paired with specific clinical contexts, indicating that respondents could envision practical applications. Overall, healthcare-professionals primarily evaluate new technologies based on how they support clinical decision-making and workflow efficiency. Notable verbatim responses include: “Interesting. Easy to use. Something new, definitely helpful for clinicians.” The overall high frequency of positive mentions regarding ease of use indicates that the solution has succeeded in creating an accessible interface, with quick access to patient data being a particularly valued feature. Overall, clinician assistance and workflow optimization emerged as the most frequently mentioned benefits. Finally, the positive mentioning of patient education features suggests that the solution is seen as valuable also beyond just clinician use.

The challenges identified in AQ2 reveal important implementation considerations that must be addressed. The pattern suggests that while the technical functionality is appreciated, the practical deployment will face social, workflow, and technical integration challenges. In addition to concerns related to user adoption, particularly for elderly patients, concerns about handling complex cases, suggest that while the solution works well for routine scenarios, it may need enhancement and more AI services for more complicated clinical situations. The clear mention of “more AI”, indicates that respondents see potential for more advanced and AI-supported decision support. Care delivery impact concerns also suggest issues related to how the technology is introduced and how it might change established care processes. The technical issues can be grouped as Audio Quality Issues (e.g., “Unclear speech/voice”, “Echo problems”, “Poor sound quality”), and Technical Limitations (e.g., “Small screen size”, “Movement can be rigid”, “Limited and supervised integration with existing systems”) While the technical issues are described with concrete, direct language, human interaction concerns are expressed with more nuanced, emotional vocabulary with consistent focus on patient impact.

The improvement suggestions in AQ3 highlight that healthcare professionals already envision an expanded role for the technology beyond its current implementation. Suggested improvements focus on enhancing existing functionalities while expanding into new applications, showing a forward-looking perspective from healthcare professionals. The language used in suggesting improvements (i.e., “AQ3”) is highly positive and reveals a forward-thinking, solution-oriented mindset. Language clarity improvements and interface optimization are the most frequently suggested enhancements, indicating that communication quality remains a priority. The mentioning of research applications, clinical trials and specialty applications (i.e., oncology, internal medicine) indicate that respondents see potential beyond current use cases. The mentioning of “more” AI implementation suggestions directly addresses the limitations mentioned in AQ2, showing that expert-users see AI as a logical next step. Finally, the interest in non-medical functions, such as informed consent, suggests potential for broader applications within the healthcare environment.

## Discussion

### Insights on acceptance and usability

In the study we examined the feasibility and acceptance of implementing a CDSS delivered through a SAR during medical grand rounds. The evaluation involved two distinct groups of healthcare professionals. The longitudinal intervention group comprised younger clinicians (mean age 32.5 years, SD = 2.78) primarily from surgical departments, with thoracic surgery representing the majority (63.5%). The single-session evaluation group represented a broader age range and more diverse specialties, with a mean age of 47.8 years (SD = 10.4) and representation from oncology (28.1%), surgical departments (18.8%), internal medicine (15.6%), emergency and intensive care (9.7%), pediatrics (6.5%), and other clinical departments (28.1%).The implementation demonstrated both feasibility and strong user acceptance, particularly among those users that were exposed to the intervention for longer period.

These findings strongly support feasibility and acceptance, thus gives a strongly positive answer to the research question 1, “Is it feasible and acceptable to implement a robot-delivered CDSS during medical Grand Rounds?”

Moreover, the findings extend previous research on the role of socially assistive robots in healthcare. The positive reception of Frida as an interface for medical system aligns with Rossi et al.'s^15^ and Blavette et al.'s^16^ findings on the acceptability of robots in clinical settings. The qualitative feedback suggests that the robot facilitated better access to clinical information while maintaining efficient workflows demonstrates the potential of SARs to enhance, rather tan disrupt, clinical practices.

Our findings on the feasibility and acceptance of implementing a CDSS delivered through a SAR during medical grand rounds align with and extend the protocol described by Mlakar et al.^27^ for evaluating the impact of integrating a CDSS and a socially assistive humanoid robot into grand rounds. Their study focuses on setting up study protocols and comparing standard care with CDSS-assisted care in terms of health outcomes. Our study primarily examined the feasibility and acceptance of the CDSS-SAR system. This difference provides complementary insights into the implementation of CDSS in clinical settings.

The positive reception of our system by healthcare professionals, particularly in terms of improved access to clinical information and workflow efficiency, supports the potential benefits hypothesized in Mlakar et al.'s protocol. This study goes further by demonstrating the evolution of user engagement over time. The high usability scores reported in our longitudinal group suggest that with extended exposure, many of the initial barriers to adoption can be overcome, an aspect that could inform future studies following Mlakar et al.'s protocol.

The comparison between single-session and longitudinal users reveals important insights about the adoption process. With the findings suggesting development of stronger habits and routines over time, the results confirm hypothesis H3. In order, however, to answer the research question 2, “How do user perceptions and system effectiveness differ between single exposure and longitudinal use?”, the following subsections discuss several aspects of acceptance and perceived usability in more detail.

### Performance and effort expectations

The acceptance patterns across both user groups revealed nuanced insights into the system's perceived utility and ease of use. The longitudinal intervention group demonstrated consistently high UTAUT2 scores, with Performance Expectancy (initial M = 4.11, final M = 4.00, t(5) = .38, p = .721, d = −.19) and Effort Expectancy (initial M = 4.33, final M = 4.28, t(5) = .21, p = .842, d = −.10) maintaining strong levels throughout the study period. In contrast, single-session users reported lower but still positive scores (Performance Expectancy: M = 3.45, SD = .99; Effort Expectancy: M = 3.58, SD = .77), with the differences between groups reaching statistical significance for Effort Expectancy (p = .027) but not for Performance Expectancy (p = .123).

**Figure 5.**
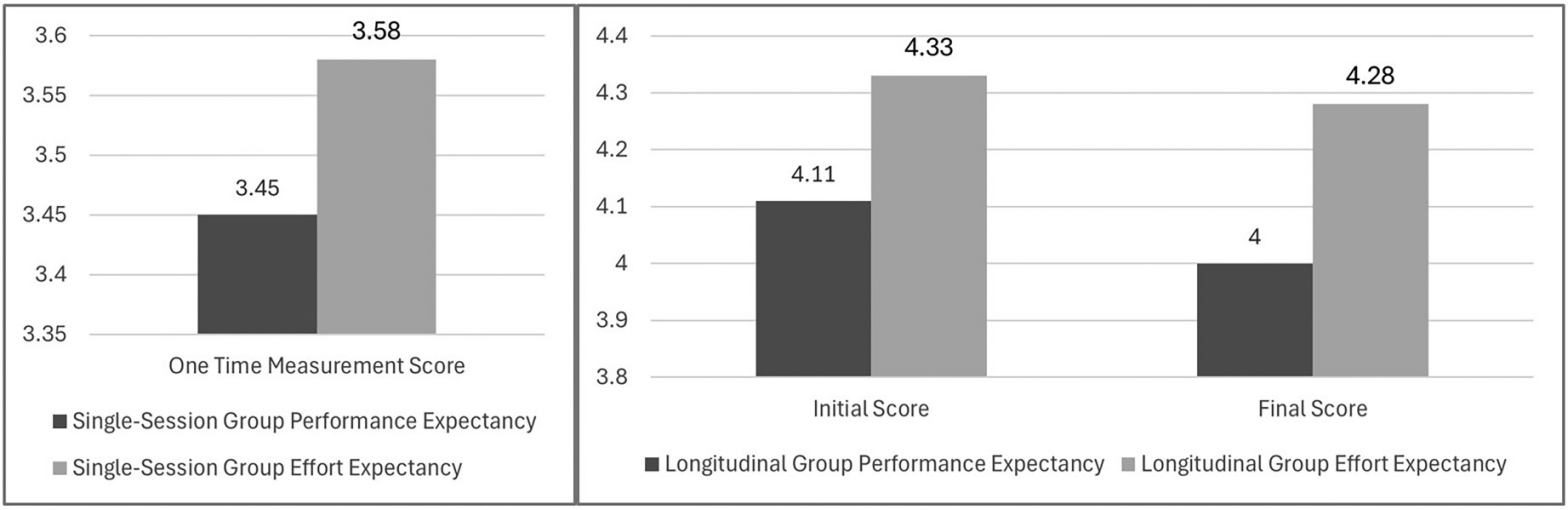
Performance expectancy and effort expectancy scores for the single-session and longitudinal group.

Figure 5 represents changes in Performance Expectancy and Effort Expectancy scores for the longitudinal and single-session group over time. The minimal decline suggests sustained perceived value and usability, while the significant difference in Effort Expectancy between groups highlights the impact of extended exposure on ease of use.

The higher scores in the longitudinal group align with Sutton et al.'s^1^ findings on Clinical Decision Support Systems’ (CDSS) role in supporting rapid clinical decision-making, while also addressing Hak et al.'s^2^ concerns about decision-making complexity in time-constrained environments. The minimal decline in both metrics over time (−0.11 for Performance Expectancy, −0.05 for Effort Expectancy) suggests that the system maintained its perceived value and usability, contradicting the typical pattern of declining satisfaction, as noted by Chen et al.'s (2023)^3^ in their research on healthcare technology adoption.

The significant difference in Effort Expectancy between single-session and longitudinal users (initial: p = .027; final: p = .041) provides valuable insights into the system's learning curve. While single-session users found the system moderately easy to use (M = 3.58), extended exposure substantially improved this perception (M = 4.33), supporting Laka et al.'s^8^ emphasis on the importance of adequate training and familiarization periods in CDSS implementation. While the results, support hypothesis H1, that clinical will accept the proposed solution, they also suggest that extended exposure can lead to notably higher acceptance levels, particularly in terms of perceived ease of use.

### System usability and integration

The System Usability Scale (SUS) scores demonstrated a difference between longitudinal and single-session users. The longitudinal group maintained exceptionally high scores (initial M = 84.58, SD = 10.42; final M = 82.08, SD = 13.91), significantly exceeding both the standard acceptability threshold of 68 and typical scores for digital health applications reported by Papadopoulos et al.^4^ In contrast, single-session users reported lower but still acceptable scores (M = 68.96, SD = 9.72), just meeting the standard usability threshold. These SUS scores align with Papadopoulos et al.'s^4^ findings regarding initial interactions with healthcare technologies, where users typically report moderate usability levels as they navigate new interfaces.

**Figure 6.**
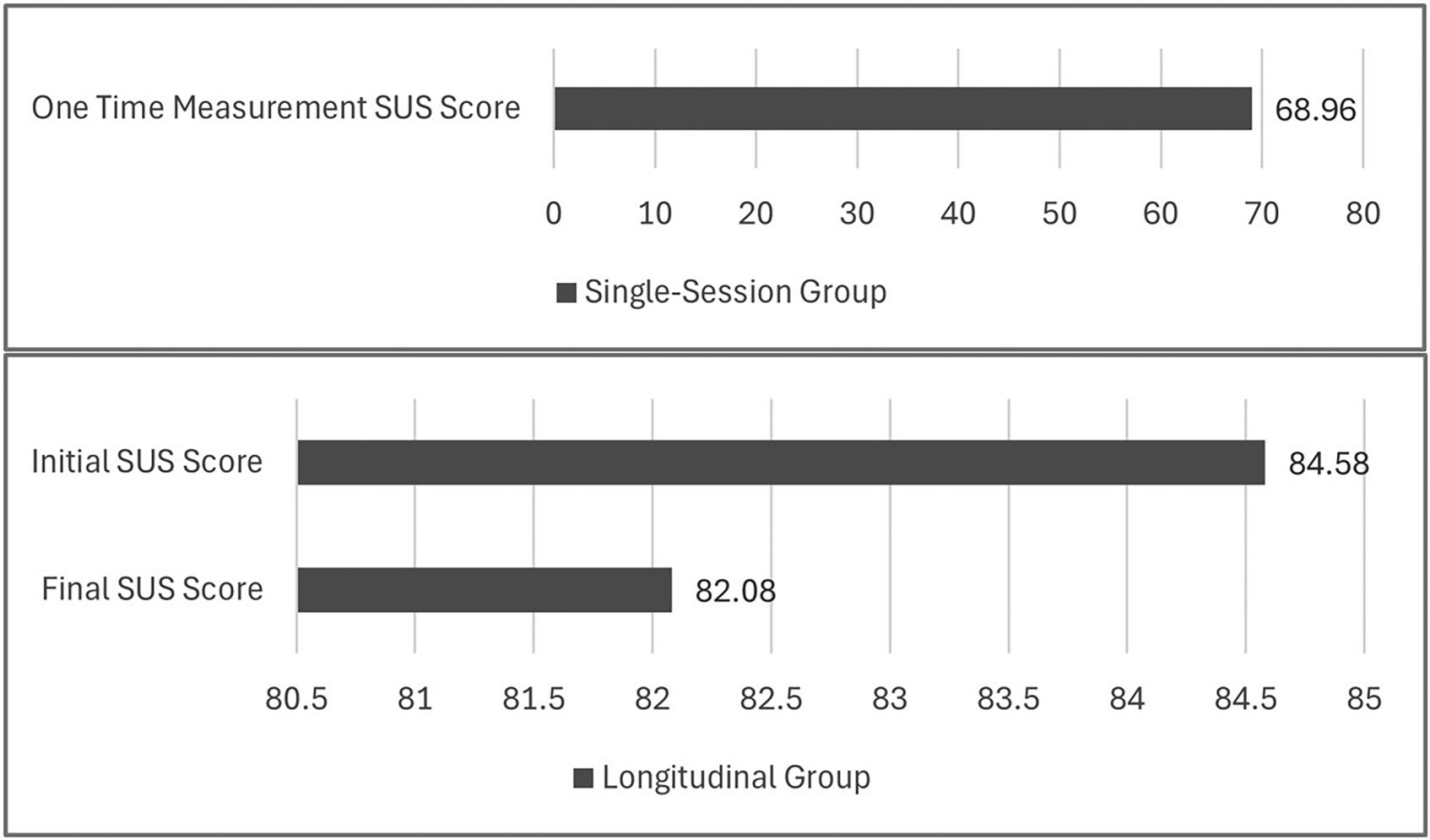
SUS scores between single-session and longitudinal group.

Figure 6 shows comparison of SUS scores between longitudinal and single-session group. The longitudinal group maintained significantly higher usability perceptions throughout the study, while the single-session group's scores remained at the threshold level.

The consistently high SUS score in the longitudinal group and positive feedback on efficiency and clinical information access support our initial hypothesis H2. However, the results for the single-exposure group are on the border of acceptance. The significant difference between groups (p = .001 for initial comparison, p = .008 for final comparison) suggests that extended exposure substantially enhanced usability perceptions. These results suggest that some of the concerns highlighted by Meunier et al.^6^ regarding CDSS adoption barriers were successfully mitigated in the longitudinal group. While the result is not completely aligned with the Chen et al.'s^3^ observation about declining satisfaction with healthcare technologies over time, the stability of the longitudinal group's high SUS scores over time (only a 2.5-point decrease) further support the claim on a successful integration into clinical workflows. Namely, the sustained high usability was, identified as critical factor by multiple studies (Sutton et al.; Laka et al.)^1,8^ for successful CDSS implementation.

### Evolution of user engagement

The combination of increasing Hedonic Motivation and strengthening Habit scores, observed in the study, indicate a successful integration of technology into clinical practice, addressing key concerns raised by Chen et al.^3^ about healthcare technology sustainability.

The analysis of user engagement patterns revealed, particularly interesting patterns in Hedonic Motivation and Habit formation. The longitudinal group showed notable increases in Hedonic Motivation (from M = 3.72 to M = 4.17, t(5) = −2.17, p = .082, d = 1.04) and Habit (from M = 3.06 to M = 3.56, t(5) = −1.42, p = .215, d = .86) over time. In contrast, single-session users reported lower Habit scores (M = 2.72, SD = .54) but relatively high Hedonic Motivation (M = 3.95, SD = .87), suggesting immediate engagement but limited routine integration. This pattern shows a strong initial appeal and increasing scores in longitudinal group indicate sustained and growing engagement. The observation is well aligned with Lim et al.'s^12^ findings about the development of positive interaction patterns with robotic systems over time.

**Figure 7.**
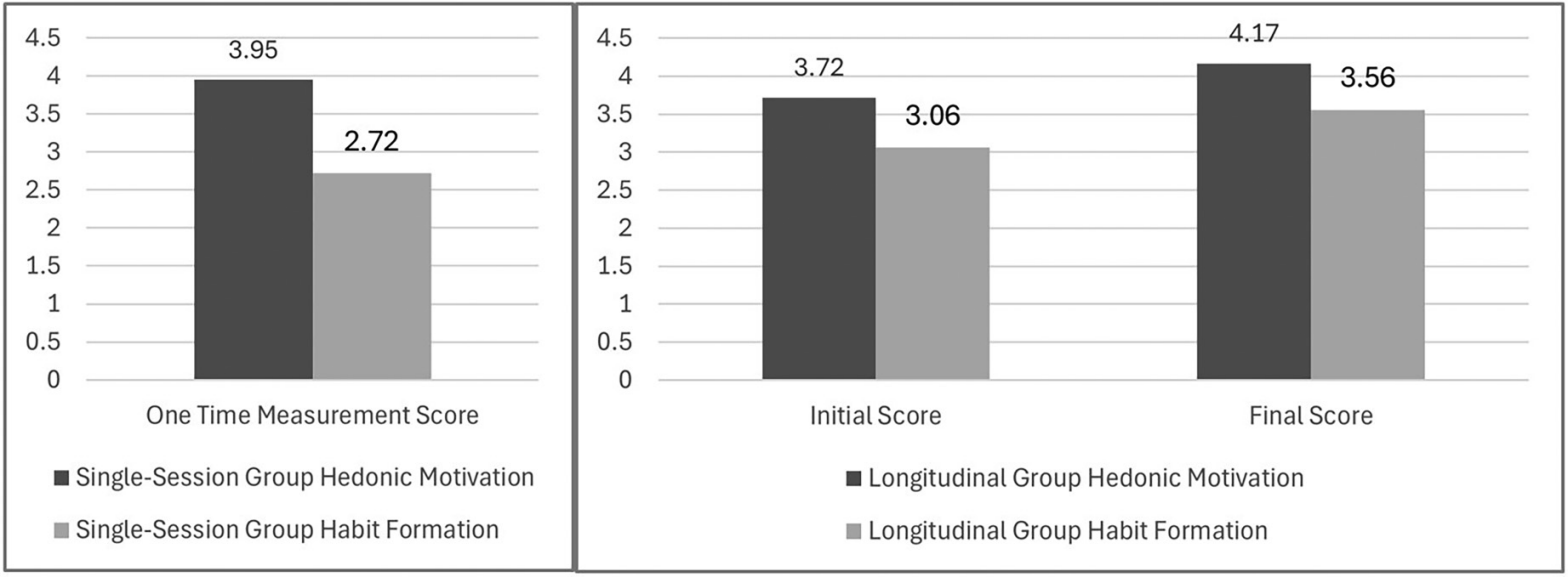
Trends in hedonic motivation and habit scores for single-session and longitudinal group.

Trends in Hedonic Motivation and Habit scores are represented in Figure 7. The longitudinal group showed an increase in both metrics, indicating growing engagement and routine integration, whereas single-session group reported only moderate habit formation.

Moreover, the substantial difference between single-session (M = 2.72) and longitudinal users’ final measurements (M = 3.56) reached statistical significance (p = .003). This difference supports Blavette et al.'s^16^ observations about the importance of repeated interactions in developing comfortable routines with healthcare technologies. The changes in Habit in the longitudinal group, i.e., M = 3.06 after initial exposure and M = 3.56 at the final exposure, are aligned with habit development, as proposed by the Kraus et al.'s^10^ model of technology adoption in healthcare settings, with three distinct phases, (1) Initial familiarization, (2) Integration period, (3) Established routine.

### Social influence and facilitating conditions

The analysis revealed patterns in Social Influence between longitudinal and single-session users. The longitudinal group showed a moderate decline in Social Influence scores (from M = 3.39 to M = 2.89, t(5) = 1.17, p = .296, d = −.61), while single-session users demonstrated moderate initial scores (M = 3.10, SD = .69). This contrast provides important insights into how social factors affect technology adoption over time. The declining Social Influence scores in the longitudinal group, despite non-significance, suggest an important shift in user behavior that aligns with Chen et al.'s (2023) observations about technology adoption patterns. The moderate effect size (d = −.61) indicates that users became less dependent on colleagues’ opinions over time, potentially reflecting increased confidence, development of personal usage patterns and the transition from socially-motivated to individually-motivated use of CDSS and Socially Assistive Robots (SARs).

Facilitating Conditions showed a pattern of the longitudinal group maintaining relatively stable scores (initial M = 3.94, final M = 3.67, t(5) = 1.05, p = .341, d = −.53) compared to lower scores in the single-session group (M = 3.21, SD = .61). The significant difference between groups in initial measurements (p = .008) suggests the importance of organizational support structures in early adoption phases.

**Figure 8.**
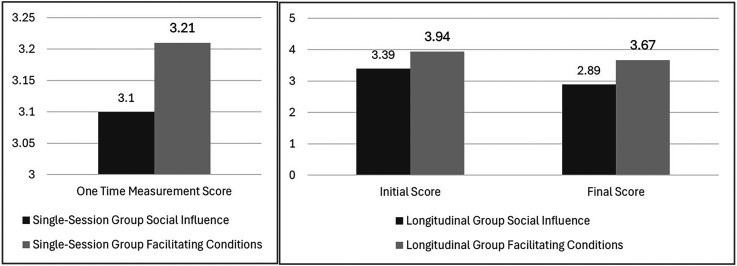
Comparison of social influence and facilitating conditions scores between the single-session and longitudinal group.

Figure 8 represents comparison of Social Influence and Facilitating Conditions scores between the longitudinal and single-session groups. The decline in Social Influence scores for the longitudinal group suggests increased self-reliance, while the stable Facilitating Conditions scores indicate sustained organizational support.

The observed patterns align with Laka et al.'s^8^ findings about the critical role of organizational support in CDSS implementation. The initial high scores indicate adequate technical infrastructure, while stable scores suggest sustained support quality. The moderate decline in longitudinal scores may reflect reduced need for technical assistance and the development of self-sufficiency. Higher scores among longitudinal users suggest successful integration into standard workflows, alignment with workflow requirements and successful adaptation to organizational processes.

The decreasing reliance on social influence over time suggests that as users become more familiar with the system, they develop their own opinions and usage patterns, potentially leading to more personalized and efficient use of technology. Meanwhile, the sustained importance of facilitating conditions underscores the need for ongoing organizational support to ensure successful long-term adoption and integration of CDSS and SAR systems into clinical workflows.

### Implementation challenges and solutions

The qualitative feedback revealed several implementation challenges that echo those identified in previous literature. Audio quality issues and system integration problems emerged as primary concerns, reflecting the integration challenges discussed by Laka et al.^8^ Feedback about screen size and interface design aligns with Meunier et al.'s^6^ emphasis on user-friendliness in CDSS adoption. However, the positive responses regarding efficiency and time management suggest that our system successfully addressed some of the workflow disruption concerns highlighted by Meunier et al.^6^ The expressed need for better integration with existing hospital systems mirrors the interoperability challenges discussed in previous studies (Chen et al.; Laka et al.).^3,8^ Finally, positive feedback about time savings and improved access to clinical information supports Sutton et al.'s^1^ findings on CDSS benefits for clinical efficiency.

### Limitations

As with most studies, also this study does not come without limitations. The small sample size in the longitudinal group (n = 6) likely contributed to Type II errors, where real effects may have gone undetected. This limited statistical power and may have contributed to the lack of statistical significance in some comparisons. The study was conducted at a single medical center, potentially limiting generalizability. Moreover, the six-month duration, while sufficient to observe initial adoption patterns, may not capture long-term integration effects. The dropout rate (25%) in the longitudinal group, though modest, might indicate selection bias toward more technologically accepting participants. Finally, the gender distribution (3:1 male-to-female ratio in the longitudinal group; 59% male in the single-session group) reflects typical gender ratios in surgical specialties. While this distribution may affect generalizability, our finding of no significant gender-based differences in system acceptance is consistent with recent studies on healthcare technology adoption.^7^ Although the system demonstrated promising usability and acceptance metrics, it is important to note that none of the observed changes reached statistical significance, likely due to the small sample size (n = 6). Further research with larger samples is needed to draw more definitive conclusions about the system's effectiveness.

Possible factors contributing to dropout were time constraints specific to certain specialties, workload differences across departments and varying levels of institutional support for research participation. To address these issues in future studies, we recommend conducting exit interviews with dropouts to understand their reasons, implementing targeted retention strategies for at-risk departments and ensuring equal support and incentives across all participating specialties. Participants participated in weekly grand rounds over the six-month period.

### Clinical implications

These results contribute to the growing body of evidence supporting the feasibility of robot-assisted CDSS implementation in clinical settings, while also highlighting important considerations for future deployments. The consistently high Behavioral Intention scores indicate strong potential for long-term adoption, addressing the sustainability concerns raised in digital health implementation literature. However, we acknowledge that sustainability extends beyond initial adoption and involves factors such as continued engagement, system adaptability, and organizational support. While our study primarily focuses on adoption potential, future research should explore these additional elements to provide a more comprehensive assessment of sustainability. This study also provides several practical contributions for healthcare organizations implementing CDSS through socially assistive robots. Firstly, the study reveals the positive correlation between exposure time and system acceptance, further emphasizing the importance of structured implementation programs with adequate training and support. Secondly, it identifies technical challenges and the need for robust IT infrastructure and support systems in healthcare facilities implementing similar solutions. Finally, the study shows that successful integration of CDSS through a SAR interface demonstrates a viable approach to enhancing clinical decision-making while maintaining workflow efficiency.

## Conclusion

This study highlights the feasibility and acceptance of integrating CDSS with SARs in clinical settings during hospital grand rounds. By using technologies, such as, the HL7 FHIR framework and humanoid robots, the intervention showed great potential to improve clinical workflows, enhance decision-making, and engage clinicians making it easier for them to access critical information quickly. The findings showed that extended use of the CDSS improved usability, familiarity, and integration into daily routines. Clinicians with longer exposure scored higher in areas, such as, effort expectancy, habit, and system usability compared to those with only a single session. Qualitative feedback also showed that the system helped save time and improved access to information, though some areas, such as robot Frida audio quality and broader integration, would need improvement. Overall, the solution provided a user-friendly CDSS dashboard over the tablet device that bridged the gap between clinicians and digital systems, minimizing workflow disruptions and encouraging consistent use. The key to- successes-factors appear to be the combination of intuitive interaction, workflow integration, and sustained engagement over time. Namely the SAR interface delivered provided a more natural interaction medium, addressing the user interface concerns highlighted by Meunier et al. Moreover, the integration of HL7 FHIR standards facilitated seamless data exchange even with the hospital's native system, resolving the interoperability challenges noted by Papadopoulos et al. Finally, the system's capacity to present contextually relevant information during grand rounds addressed the workflow integration concerns identified in multiple studies. Overall, this research offers valuable insights into the role of SARs in healthcare, showing how they can support clinical decision-making and transform traditional CDSS into more interactive and efficient tools. Future efforts should address technical issues, maybe even provide additional training for hospital staff, and explore strategies for long-term integration to fully realize the potential of SAR-assisted CDSS in diverse healthcare environments.

## Supplemental Material

sj-doc-1-dhj-10.1177_20552076251339012 - Supplemental material for Feasibility of a computerized clinical decision support system delivered via a socially assistive robot during grand rounds: A pilot study Supplemental material, sj-doc-1-dhj-10.1177_20552076251339012 for Feasibility of a computerized clinical decision support system delivered via a socially assistive robot during grand rounds: A pilot study by Valentino Šafran, Urška Smrke, Bojan Ilijevec, Samo Horvat, Vojko Flis, Nejc Plohl and Izidor Mlakar in DIGITAL HEALTH
